# Antioxidants and Anti-Inflammatory Effects in Neurodegenerative Diseases (NDs)

**DOI:** 10.3390/antiox11061172

**Published:** 2022-06-14

**Authors:** Michela Campolo, Irene Paterniti

**Affiliations:** Department of Chemical, Biological, Pharmaceutical and Environmental Sciences, University of Messina, Viale Ferdinando Stagno D’Alcontres, 31-98166 Messina, Italy

Neurodegenerative diseases (NDs) are the most common chronic neurological diseases associated with age, and they have a strong impact on the patient’s quality of life [1]. NDs include sporadic disorders of the central nervous system which involve dysfunctions in synapses, neurons, glial cells, and their networks in specific brain areas, such as the temporal, limbic and mesencephalic areas [2]. The most common NDs are Alzheimer’s disease (AD), Parkinson’s disease (PD), amyotrophic lateral sclerosis (ALS) and Huntington’s disease (HD) [3]. Several studies have shown that oxidative stress and neuroinflammation are mechanisms that are closely related and highly involved in the progression of NDs [4,5,6,7]; thus, the study of new molecular targets and new pharmacological candidates that act by regulating these two mechanisms is a new goal in research. This Special Issue includes five articles in which the authors evaluate the anti-inflammatory and antioxidant effects of well-known molecules through both in vitro and in vivo neuroinflammatory models. An in vitro study performed by Kumar, M.R et al. [8] evaluated the antioxidant and neuroprotective effects of six different kefir water samples cultured in Malaysia on H_2_O_2_-stimulated differentiated human neuron cells. The data obtained showed that Kefir B water keeps the neuronal membrane structure intact after exposure to oxidative damage. As traumatic brain injury (TBI) represents a risk factor for the development of neurodegenerative diseases such as dementia and AD, M. Cordaro et al., demonstrated the anti-inflammatory and antioxidant effects of Hidrox^®^, a derivative obtained during the treatment of Olea europaea, in a rat model of secondary injury induced by TBI and the propagation of AD-like neuropathology [9]. In recent years, research has focused on the study of the nutraceutical properties of fermented products. In their review, Muganti R. K. et al. [10] reported in vitro and in vivo studies that best examine the neuroprotective, antioxidant, anti-inflammatory, anti-tumor and immunomodulating effects caused by the consumption of fermented foods, thus representing a promising alternative for the therapeutic management of AD.

Ibrahim Khan’s team first studied the human steroid hormone, 17 β-estradiol, in a rat model 7 days after birth, highlighting its neuroprotective and anti-inflammatory abilities against glutamate-induced reactive oxygen species (ROS) production by regulating the nuclear factor erythroid 2–related factor 2/heme oxygenase-1 (Nrf2/HO-1) antioxidant pathway and by regulating the mitogen-activated protein kinase (MAP-kinase) pathway, making it the subject of future studies in ensuring neuroprotection [11]. One of the limitations of NDs is that to date, the exact etiopathological mechanisms are still not clear. In this Special Issue, a study regarding samples of subjects with ALS conducted by Sun, H. et al., who identified pathogenic genes mainly involved in the regulation of inflammatory processes and potential markers useful in the prediction of the prognosis of ALS [12]. The review article in this Special Issue discussed the antioxidant and anti-inflammatory effects of astrocytes and their synergy with microglia and dopamine involved in the pathogenesis and pathophysiology of PD [13]. I would like to acknowledge the authors who have contributed to this Special Issue, entitled “Antioxidants and Anti-Inflammatory Effects in Neurodegenerative Diseases”, which highlighted the recent discoveries in the field of NDs, with particular attention to the involvement of neuronal oxidative stress and inflammatory processes. In addition, the anti-inflammatory and antioxidant effects of molecules, which could represent new pharmacological candidates for counteracting the progression of NDs, were investigated.
